# Therapeutic Potential of Oxiracetam in Neurocognitive Disorders: A Narrative Review

**DOI:** 10.1002/brb3.71727

**Published:** 2026-08-30

**Authors:** Hafsa Ali, Syeda Maliha, Sundas Ishtiaq, Omer Al‐Karawi, Mohammed Hammad Jaber Amin

**Affiliations:** ^1^ Sindh Medical College Jinnah Sindh Medical University Karachi Pakistan; ^2^ College of Medicine University of Mosul Mosul Iraq; ^3^ Faculty of Medicine Alzaiem Alazhari University Khartoum Sudan

## Abstract

**Purpose**: Cognitive impairment is a growing global health concern associated with aging, neurodegenerative diseases, stroke, and traumatic brain injury. Existing pharmacological treatments, including cholinesterase inhibitors and memantine, provide limited and variable benefits. This review aims to evaluate the mechanistic basis, clinical efficacy, and safety profile of oxiracetam as a potential therapeutic agent for cognitive dysfunction across a range of neurological disorders.

**Methods**: A narrative review was conducted by synthesizing available preclinical and clinical literature on oxiracetam. Mechanistic studies evaluating its pharmacodynamics, including effects on cholinergic and glutamatergic pathways, synaptic plasticity, neuroinflammation, and oxidative stress, were analyzed. Clinical evidence from trials involving patients with traumatic brain injury, stroke, vascular cognitive impairment, and dementia was reviewed.

**Findings**: Oxiracetam demonstrates multimodal mechanisms of action, including enhancement of cholinergic neurotransmission, modulation of AMPA receptors, and promotion of synaptic plasticity and long‐term potentiation. Additionally, it exhibits antioxidant and anti‐inflammatory effects contributing to neuroprotection. Clinical studies suggest potential cognitive benefits in several neurological conditions; however, results are heterogeneous, with some trials showing significant improvement and others demonstrating no superiority over placebo.

**Conclusion**: Oxiracetam is a promising neurocognitive modulator with a favorable safety profile and potential therapeutic applications across multiple neurological disorders. Nevertheless, current evidence is limited by methodological constraints and inconsistent outcomes. Further large‐scale, multicenter clinical trials are required to establish its definitive efficacy and clinical utility.

## Graphical abstract

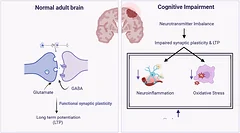

## 1 Introduction

Cognition is the ability of the brain to comprehend information and support memory, thinking, and decision‐making. A decline in this ability is called cognitive impairment, making everyday functioning difficult (Academy of Cognitive Disorders of China (ACDC) et al. 2020). Cognitive impairment becomes more prevalent with increasing age, presenting a serious risk to public health (Zhou et al. 2022). Factors like neurodegenerative diseases, cardiovascular and cerebrovascular disorders, nutritional and metabolic issues, infections, traumatic brain injuries, and substance abuse can lead to it. It is generally divided into mild cognitive impairment (MCI) and dementia (Academy of Cognitive Disorders of China (ACDC) et al. 2020; Zhou et al. 2022).

Dementia is a leading cause of cognitive decline. The number of people around the world affected by dementia is over 47 million, with close to 7.7 million new incidents per year. Alzheimer's disease is the most prevalent, while vascular dementia (VaD) takes on 20% of all dementia cases (Li et al. 2017). Another contributing factor to cognitive decline is traumatic brain injury (TBI), which affects about 50 million people yearly. It can lead to long‐term cognitive issues in about 10% of mild cases and greater than 60% of moderate‐to‐severe cases, placing stress on the healthcare system (Liu et al. 2024). There can be cognitive problems after a stroke called post‐stroke cognitive impairment, which makes recovery difficult and also decreases the quality of life (Lim et al. 2026). Together, these conditions are pressing issues for society, the economy, and the healthcare system, emphasizing the urgent need for the development of effective prevention and treatment options. While the specific biological mechanisms for each of these diseases are not fully understood, it has been shown through some studies that reduced energy metabolism, oxidative stress, mitochondrial dysfunction, and neuronal death are all related to cognitive impairment (Zhou et al. 2022).

The current treatment options for cognitive impairment include cholinesterase inhibitors (donepezil, rivastigmine, galantamine) and memantine, an NMDA receptor antagonist (Zhou et al. 2022). Cholinesterase inhibitors have shown results in improving cognition in AD, although their use in advanced stages of AD and their long‐term effects remain unclear. Their response to VaD is mixed and inadequately addressed in other neurocognitive disorder conditions. Systematic analysis of clinical data shows that memantine provides modest improvement in moderate‐to‐severe forms of AD, and a limited effect on mild‐to‐moderate forms of AD or VaD (Hugo and Ganguli 2014). Presently, there are no effective treatment options for cognitive impairments following traumatic brain injuries (TBIs) or for vocational rehabilitative services post‐TBI (Liu et al. 2025). Similarly, there aren't many drug choices available to treat post‐stroke cognitive impairment (Lim et al. 2026). The cost of post‐stroke cognitive rehabilitation places an enormous burden on the family and society (Fu et al. 2024). Overall, the current treatment options available for cognitive impairment for all neurological conditions remain limited. Thus, there is an immediate and urgent need for new treatment options that improve cognitive functioning, support brain protection, and act on the underlying causes of neurological disorders.

Oxiracetam is a nootropic drug that belongs to the racetam family and has shown potential as a neurocognitive modulator that can provide therapeutic benefit to a broad range of brain disorders, including anxiety, stroke, degenerative neurological disorders, cognitive deficits, including memory loss, and seizure disorders (Xu et al. 2019). Clinical studies have demonstrated that oxiracetam can improve cognitive functioning in geriatric patients, inhibit the release of inflammatory cytokines after stroke, and improve memory in patients suffering from VaD and cerebral hypoperfusion (Zhang et al. 2020). These observations are consistent with the possibility that oxiracetam represents a viable therapeutic option for cognitive dysfunction/disorders across a spectrum of neurological conditions, wherein previously available treatments have had limited effect.

The purpose of this article is to present the mechanistic and clinical evidence to date regarding the use of oxiracetam as a neurocognitive modulator within the various categories of neurological disorders, to evaluate its clinical efficacy and safety, and to provide insights into potential future research directions in the field of cognitive neuropharmacology.

## 2 Methodology

A comprehensive literature search was conducted using PubMed, Scopus, Embase, and Cochrane from inception till April 2025. Search terms and keywords included “Oxiracetam” OR “4‐hydroxy‐2‐oxo‐1‐pyrrolidine acetamide” OR “ORT” AND “Neurocognitive Disorders” OR “Cognitive impairment” OR “Traumatic Brain Injury” OR “Neurodegenerative disorders” OR “Vascular Cognitive Impairment” OR “Dementia.” Included articles were clinical trials, published in English on the use of oxiracetam in various neurocognitive conditions. Studies on any other disorders were excluded.

## 3 Chemical Structure and Pharmacologic Profile

### 3.1 Racetam Class Overview

Piracetam (2‐oxo‐1‐pyrrolidine acetamide), also referred to as a nootropic, is a derivative of the neurotransmitter gamma‐aminobutyric acid (GABA). It belongs to the racetam class (Verma et al. 2018). Other compounds of the racetam class include oxiracetam, pramiracetam, etiracetam, nefiracetam, and aniracetam. Although it is assumed that the racetam derivatives are similarly functioning, the efficacy and strength of them is highly varied in each of the compounds (Malík and Tlustoš 2022).

Oxiracetam (4‐hydroxy‐2‐oxo‐1‐pyrrolidine acetamide) is a derived form of piracetam (Wang et al. 2016; Zhang et al. 2024). Oxiracetam is a chiral compound because it has a chiral center at carbon number four of the 2‐pyrrolidone ring; as a result, oxiracetam can exist as either (S)‐or (R)‐enantiomers. The form that is marketed is a racemic mixture composed of equal amounts of both enantiomers. According to research, the more pharmacologically active isomer is (S)‐oxiracetam, also known as L‐oxiracetam. It was more successful than the (R)‐form in improving long‐term potentiation, boosting calcium uptake in neuronal cells, and restoring scopolamine‐induced amnesia in rats, according to early research. Recent research also indicates that (S)‐oxiracetam is mostly in charge of enhancing memory and learning, decreasing pathological damage, and boosting cerebral blood flow (Wang et al. 2016; Zhang et al. 2024).

### 3.2 Pharmacokinetics

Physicochemical characteristics of enantiomers are often very similar; however, drug pharmacokinetics and biological activity can differ substantially, and thus can lead to different pharmacodynamic potential as well as different adverse effects. The pharmacokinetics of (S)‐oxiracetam have been largely studied in animals, with limited human data and only one investigation of its intravenous use. According to recent research, (S)‐oxiracetam is rapidly absorbed following oral administration in humans. In a single‐ascending‐dose (SAD) study of oxiracetam, the average time to reach the maximum plasma concentration (Tmax) ranged from 0.75 to 1 h. On the contrary, the multiple‐ascending‐dose study showed a higher Tmax range from 0.5 to 1.5 h (Zhang et al. 2024). A separate study performed on beagle dogs concluded that at 1.5 and 2 h post‐dose, the plasma concentrations and AUC_0_∞ of (S)‐oxiracetam were significantly higher than those of (R)‐oxiracetam. This suggests that (S)‐oxiracetam is better absorbed than (R)‐oxiracetam. Speaking generally, these observations support previous claims on racemic oxiracetam showing rapid absorption in the GI tract and systemic availability (Zhang et al. 2024). Oxiracetam can easily cross the blood–brain barrier (BBB) and concentrates at multiple sites in the brain substance, including the septum, hippocampus, cerebral cortex, and striatum (Li et al. 2017).

The elimination half‐life of the (S)‐oxiracetam for SAD ranged between 6.12 and 6.60 h. These findings are slightly longer than what has been previously reported for (S)‐oxiracetam in other test populations (approximately 4.28 to 5.21 h), and were also found to be shorter than the elimination half‐life reported for oxiracetam (approximately 8.5 h). The majority of (S)‐oxiracetam was excreted from the body primarily as the unchanged parent compound. The mean urinary excretion and fecal excretion cumulative ratios following a single oral dose of (S)‐oxiracetam were approximately 55% and 36%, respectively. The total recovery of unchanged drug in urine and feces within 48 h exceeded 90%, fulfilling conventional mass balance criteria (Zhang et al. 2024). Similar pharmacokinetic parameters were reported for both enantiomers concerning elimination half‐life, absorption half‐life, time to reach peak concentration, volume of distribution, and clearance (Wang et al. 2016). Moreover, chiral drugs often undergo enantiometric conversion in vivo, which eventually results in reduced efficacy or increased toxicity. However, after oral administration of (S)‐oxiracetam, the lowest concentrations of (R)‐oxiracetam were observed in both urine and feces, which indicates a minuscule conversion rate of (S)‐oxiracetam to (R)‐oxiracetam. This indicates negligible chiral inversion in vivo. Consequently, (S)‐oxiracetam appears to be stereochemically stable and is excreted largely as the unchanged active enantiomer (Zhang et al. 2024).

The pharmacokinetics of oral medicines may be influenced by food by impacting gastric emptying and changing the conditions in the GI tract. (S)‐Oxiracetam is absorbed almost entirely unaffected by food because of its superior solubility in water; however, high‐fat meals delay Tmax from 1 to 3 h due to their impacts on gastric emptying time. It can therefore be taken with or without food (Zhang et al. 2024). These findings indicate that (S)‐oxiracetam alone may exhibit a more advantageous pharmacokinetic profile compared to racemic oxiracetam, and using the more active enantiomer alone may improve therapeutic efficacy and reduce adverse effects (Zhang et al. 2024).

### 3.3 Pharmacodynamics

Oxiracetam enhances cognitive performance through various biochemical means that facilitate brain tissue restoration and healing (Liu et al. 2025). It is thought that it either directly or indirectly acts on central cholinergic neurons by affecting the activity of acetylcholinesterase, increasing the sensitivity of the cholinergic receptor, or increasing the production/release of acetylcholine. All three will produce an observable result with regard to cognitive function (Liu et al. 2024; Dang et al. 2024). It was noted in research on mice that oxiracetam was able to stimulate cholinergic fibers in the cerebral cortex and the hippocampus, hence increasing the release of acetylcholine and supporting neural plasticity (Liu et al. 2025). Another separate study showed that high‐affinity choline uptake was promoted by oxiracetam in the hippocampus of rats, providing protection against scopolamine and electroshock‐induced depletion of acetylcholine in the brain. Consequently, it is safe to say that oxiracetam improves and supports cholinergic function in the brain (Hokonohara et al. 1992).

The ionotropic glutamate receptor, AMPA (α‐amino‐3‐hydroxy‐5‐methyl‐4‐isoxazolepropionic acid), is involved with rapid excitatory communication within the brain by allowing the flow of sodium (Na^+^) and potassium (K^+^), and keeping the resting membrane potential stable enough for neurons to carry out their job. Current research indicates that oxiracetam is a positive allosteric modulator of AMPA by increasing calcium entry and the number of available AMPA binding sites. Given that AMPA receptors mediate fast postsynaptic effects of glutamate, oxiracetam's ability to modulate AMPA may have a substantial impact on enhancing synaptic plasticity (Li et al. 2017).

Glutamate is the major excitatory amino acid in the brain (Li et al. 2017). Oxiracetam has been shown to enhance daily tasks and cognitive functions. It does this by stimulating glutamate receptor activity, which creates a greater postsynaptic potential, and by repairing damaged brain function (Li et al. 2020). Research demonstrates that (S)‐oxiracetam influences the malate‐aspartate shuttle, enhances the glutamate‐glutamine cycle, increases levels of antioxidants, and enhances ATP production. Therefore, these findings demonstrate that (S)‐oxiracetam could reduce neuron death through antioxidant enhancement, reduction of excitatory neurotoxicity, and enhancement of energy metabolism. After 2‐VO surgery, lower levels of glutamine, glutamate, aspartate, and N‐acetylaspartate (NAA) were found. However, when (S)‐oxiracetam or oxiracetam was given after the surgery, these levels were restored, suggesting that both drugs may influence glutamate/aspartate‐related energy metabolisms (Li et al. 2017).

## 4 Mechanistic Basis for Neurocognitive Modulation

Oxiractem promotes multiple neurocognitive pathways, such as long‐term potentiation (LTP), excitotoxicity, and inflammatory pathways (Table 1).

**TABLE 1:** Mechanisms of action of oxiracetam.

Mechanism	Description	Clinical relevance
**Cholinergic modulation**	Enhances acetylcholine release, receptor sensitivity, and cholinergic transmission	Improves memory, learning, and attention
**Glutamatergic modulation (AMPA receptors)**	Acts as a positive allosteric modulator, increasing synaptic transmission	Enhances synaptic plasticity and cognitive processing
**Synaptic plasticity & LTP**	Promotes long‐term potentiation and PKC activity in the hippocampus	Supports memory consolidation and learning
**Anti‐excitotoxic effects**	Reduces glutamate accumulation and calcium‐mediated neuronal damage	Protects neurons in ischemia and TBI
**Antioxidant activity**	Reduces ROS and increases SOD1/SOD2 levels	Prevents oxidative neuronal injury
**Anti‐inflammatory effects**	Suppresses COX‐2, IL‐1β, NLRP3 pathways	Reduces neuroinflammation post‐injury
**Energy metabolism enhancement**	Improves ATP production and mitochondrial function	Supports neuronal survival and function
**Blood–brain barrier protection**	Preserves tight junction proteins and reduces edema	Limits secondary brain injury

### 4.1 Synaptic Plasticity and LTP Enhancement

In long‐term potentiation (LTP), a short high‐frequency stimulation at an excitatory neuronal synapse effectively prompts a constant increase in the strength of the synapse (Rodgers et al. 2015). The LTP phenomenon is hence associated with cognitive functions; consequently, a decline in LTP can strongly influence memory and learning. Oxiracetam, particularly in the hippocampal circuits, has been shown to enhance LTP. In simple terms, LTP depends mainly on the interaction among excitatory receptors, specifically the N‐methyl‐D‐aspartate (NMDA) receptors, and their respective excitatory neurotransmitters. The synthesis of both these elements decreases in chronic cerebral hypoperfusion (CCH), owing to a reduction in blood and oxygen supply. Administration of oxiracetam promotes metabolism, enabling neurons to maximize the synthesis of excitatory receptors and neurotransmitters in hypoperfused conditions, thus enhancing LTP, which would ultimately improve cognition (Yao et al. 2016).

Adding on, the hippocampal protein kinase C (PKC) activity is also promoted by oxiracetam, and since PKC plays a role in synaptic plasticity and memory consolidation, improvements are expected. Studies have demonstrated that oxiracetam specifically increases membrane‐bound PKC levels in the hippocampus, an effect that correlates with substantial improvements in learning task performance (Youn et al. 2023).

### 4.2 Modulation of Excitotoxicity

Excitotoxicity results from extracellular accumulation of glutamate or other excitatory neurotransmitters, leading to excessive stimulation of NMDAR, calcium overload, and neuronal death. It can result from increased glutamate release in the synaptic cleft, impaired uptake due to disruption in excitatory amino acid transporters (EAAT1 and EAAT2), or decreased levels of NAAG (N‐acetylaspartylglutamate) (Marcuzzo et al. 2026). Rats treated with oxiracetam showed reduced levels of glutamate and significantly improved GABA levels; thus, it protects the brain from glutamate‐mediated excitotoxic damage (Paudel et al. 2020). Additionally, enhancement of neuronal metabolism and high‐energy phosphate availability supports ATP‐dependent ion regulation, thereby limiting calcium‐mediated excitotoxic damage (Yao et al. 2016).

### 4.3 Anti‐inflammatory and Antioxidant Effects

Oxidative stress and neuroinflammation are closely linked processes that contribute to secondary neuronal damage following central nervous system injury. Oxidative stress results from an imbalance between the production of reactive oxygen species (ROS) and the capacity of antioxidant defenses to neutralize them. Disruption of this equilibrium leads to neuronal damage and promotes lipid peroxidation, protein misfolding, and DNA damage (Kurhaluk et al. 2025). Antioxidant mechanisms normally counteract these effects by scavenging ROS and maintaining neuronal integrity (Lee et al. 2020); however, excessive inflammation following injury can overwhelm these protective systems, underscoring the need for therapeutic strategies that mitigate oxidative stress–mediated damage.

Studies show that oxiracetam can affect both oxidative stress and inflammation in the brain. In traumatic brain injury (TBI) models, oxiracetam significantly reduced intracellular ROS accumulation in the brain and increased natural antioxidant enzymes like superoxide dismutase‐1 and ‐2 (SOD1/SOD2), thus helping the brain restore its natural defense system. At the same time, oxiracetam limited neuroinflammatory signaling and apoptotic neuronal loss by suppressing important inflammatory mediators involved in inflammasome activation, such as COX‐2, NLRP3, caspase‐1, and IL‐1β. Transcriptomic and pathway analyses indicate that oxiracetam affects multiple inflammation‐related signals in the brain, including important pathways like JAK‐STAT and PI3K‐Akt. This implies that oxiracetam controls immune responses in several ways, not just through its antioxidant activity, a concept that is further discussed in the section that follows. The net results from these effects in experimental models were brain protection from cortical damage, reduced edema of the brain, and enhanced neurological and cognitive outcomes. This supports the idea that oxiracetam limits secondary brain injury by tackling oxidative stress and inflammation together (Youn et al. 2023).

### 4.4 Neuroprotection in Ischemic and Traumatic Injury Models

Oxiracetam demonstrates neuroprotective effects in a variety of injury models through coordinated regulation of transcriptional networks, preservation of structural integrity, and maintenance of blood–brain barrier function (Yao et al. 2016; Youn et al. 2023). In CCH, it prevents white matter lesions, which are particularly vulnerable to damage due to their deep location. It also improves dendritic spine formation, as shown by restored levels of brain‐derived neurotrophic factor (BDNF) and preserved activity of CREB (Yao et al. 2016). After an ischemic stroke, S‐oxiracetam may aid cognitive recovery by influencing α7 nicotinic acetylcholine receptors and related signaling pathways such as PI3K/Akt/GSK3β. These distinct, stereospecific effects set it apart from the racemic form and suggest it could be useful in developing more targeted treatments (Fan et al. 2018). Furthermore, oxiracetam helps reduce vasogenic edema and limits the entry of peripheral immune cells into the brain by preserving tight junction proteins, as reflected by decreased Evans blue leakage (Huang et al. 2017). Additionally, the substance encourages neuroprotective microglial polarization, which causes resident immune cells to change from pro‐inflammatory to reparative phenotypes that aid in tissue healing rather than prolonging damage (Wang et al. 2021).

A study conducted by Youn et al. (2023) explains the molecular mechanism through which oxiracetam ameliorates cognitive impairment in TBI. Oxiracetam exerts its neuroprotective actions through regulation of the competing endogenous RNA (ceRNA) network, which modulates gene expression by allowing lncRNAs to sponge miRNA, thus enabling messenger RNA (mRNA) to produce proteins, which can influence the brain injury process. The prolactin receptor (Prlr) gene and cell cycle regulator genes such as Cdkn1a, associated with the PI3K‐Akt pathway, exhibited significant changes after oxiracetam treatment. The PI3K‐Akt pathway regulates memory and cognitive functions by playing a major role in synaptic loss, autophagy interruption, cognitive decline, and initiating neuroinflammation after brain injury. Studies have shown that inhibition of this pathway would improve neuroinflammation post‐TBI. Prlr is also involved in another key signaling pathway (JAK‐STAT) that affects cognition. The JAK2/STAT3 pathway plays an important role in apoptotic responses post‐TBI. Western blot analysis also revealed that protein levels of p‐JAK2, p‐STAT3, p‐PI3K, and p‐Akt were decreased in oxiracetam‐treated rats. Increased expression of Cdkn1a after TBI, normally quiescent in post‐mitotic neurons, is also restored by oxiracetam, preventing pathological cell cycle re‐entry, which would ultimately result in neuronal death. Oxiracetam also seems to modulate claudin‐1 (Cldn1), a tight junction protein critical for blood–brain barrier integrity, thus minimizing inflammation. These findings suggest that the neuroprotective effects of oxiracetam in TBI models are via modulation of inflammatory and immune response pathways (Wang et al. 2025).

## 5 Clinical Evidence Across Neurological Disorders

Clinical evidence on oxiracetam mainly focuses on disorders that result in cognitive dysfunction as a sequel, such as stroke, traumatic brain injury (TBI), vascular cognitive impairment, and dementia syndromes (Table 2).

**TABLE 2:** Summary of clinical evidence of oxiracetam.

Condition	Sample size	Study design	Intervention	Comparator	Primary outcome	Key findings	Main limitations	Conclusion
**Traumatic brain injury (TBI)**	591 patients (LOCATE trial)	Multicenter Phase III clinical trial	L‐oxiracetam (4 g) and Oxiracetam (6 g)	Placebo	Change in LOCTA score from baseline to 90 days post‐treatment	Improved LOTCA scores; better cognition vs. placebo (L‐oxiracetam superior)	High patient withdrawal and no assessment of the outcome domains of headache, mood, and sleep	Promising efficacy in cognitive recovery
**TBI (animal study)**	27 healthy rat models	Preclinical study	Oxiracetam	Physiological saline injection	Neurological function measured by Gracia scores	Reduced lesion size, inflammation, improved neurological function	Not specified	Supports neuroprotective role
**Stroke (PSCI)**	500 patients	Multicenter, double‐blind, placebo‐controlled trial	Oxiracetam 800 mg	Placebo	Changes in MMSE and CDR‐SB from baseline to week 36	No significant superiority vs. placebo	Actigraphy data were missing in 13% due to device failure, gender imbalance as women represented 20% of cohort, and limited sensitivity of MMSE for post‐stroke cognitive deficits	Conflicting evidence
**Vascular cognitive impairment**	80 patients	Prospective study	Hyperbaric oxygen therapy combined with butylphthalide and oxiracetam 800 mg	Butylphthalide and oxiracetam only	MMSE score	Significant improvement in MMSE and reduction in TNF‐α, IL‐6	Lack of follow‐up data	Effective as adjunct therapy
**Dementia**	96 patients	Double‐blind clinical trial	Oxiracetam 1.6 g	Placebo	Cognitive function	Improved reaction time and cognition vs. placebo	Not stated	Suggests benefit in progressive dementia
**Vascular dementia (preclinical)**	40 healthy rats	Animal study	Oxiracetam	VaD (vehicle‐treated) rats	Cognitive/behavioral recovery	Restored BCL‐2/BAX balance, reduced neuronal apoptosis	Not stated	Neuroprotective potential

### 5.1 Traumatic Brain Injury (TBI)

TBI is an emerging problem causing mortality and long‐term morbidity worldwide, leading to a considerable burden on patients and health care systems (Liu et al. 2025; Mahumane and Choonara 2026). Contributing factors include externally applied forces, such as blunt force trauma, sudden acceleration, or deceleration leading to whiplash injuries, and piercing injuries that damage surrounding structures. The primary pathway of TBI injury eventually progresses to late‐onset secondary injuries, such as cognitive impairment, Alzheimer's, and other dementias (Liu et al. 2025; Mahumane and Choonara 2026). Post‐traumatic cognitive deterioration results from the destruction of the brain regions involved and aberrant neurotransmitter activity. Diffuse axonal injury (DAI), one of the most common lesions of TBI, is a significant predisposing factor for cognitive decline via axon segment disruption, impaired transport, and demyelination (Liu et al. 2025).

Wang et al. conducted a preclinical study involving 27 healthy rats, randomly assigned to three groups: sham, TBI, and oxiracetam. The trial indicated that oxiracetam‐administered rats showed some degree of restoration in neurological and motor function. Compared to the TBI group, oxiracetam rats also showed reduced levels of inflammation and enhanced management of bleeding. Quantitative analysis indicated that the size of the lesion in TBI rats was greater than that of sham, while a significant reduction in lesion volume was seen in oxiracetam‐treated rats. Based on this preclinical model study, oxiracetam exerts its effects through the TNF, mTOR, and JAK‐STAT/PI3K‐Akt signaling pathway, modulating the inflammatory cascade, as previously described (Wang et al. 2025).

In another clinical trial by Liu et al., 591 patients were randomly assigned to three groups: oxiracetam, L‐oxiracetam, and placebo. The Loewenstein Occupational Therapy Cognitive Assessment (LOTCA) score change was the primary outcome; higher scores indicated better cognitive abilities. Changes in the scores were observed over a period of 90 days. The mean score change in all three groups was as follows: 20.45 in the L‐oxiracetam group, 15.90 in the oxiracetam group, and 11.47 in the placebo group. An LS mean difference of 8.97 (*p* < 0.001) was observed between the L‐oxiracetam group and placebo, indicating much better neurological function following administration. Moreover, visual awareness, spatial orientation, motor function, thinking ability, focus, attention span, and visual motor integration were also improved by L‐oxiracetam administration (*p* < 0.05 for all) in patients (Liu et al. 2025).

### 5.2 Stroke

Stroke is one of the major causes of death worldwide. It occurs due to the blockage of blood flow to the brain (ischemic stroke), rupture of a vessel within the brain (hemorrhagic stroke), or bleeding into the subarachnoid space followed by severe headaches (subarachnoid stroke) (Ren et al. 2025). A common consequence of stroke is post‐stroke cognitive impairment (PSCI), impacting quality of life and rehabilitation. Lim et al. conducted a trial including 500 patients from university and general hospitals in South Korea. Participants were randomized into two groups: oxiracetam and placebo. Two main outcomes were measured over 36 weeks: the Mini‐Mental State Examination (MMSE) and the Clinical Dementia Rating–Sum of Boxes (CDR‐SB). Several secondary outcomes were also measured. Neurological involvement was higher in the oxiracetam group (43.2%) than in the placebo group (35.6%) in this human study. The trial reported that oxiracetam did not have superior efficacy compared to placebo (Lim et al. 2026); this result was inconsistent with previous findings of oxiracetam's superior efficacy in PSCI (Rozzini et al. 1992; Villardita et al. 1992). An MMSE score increase of 0.13 points in the oxiracetam group and 0.27 points in the placebo group in 9 months. In contrast, Rozzini et al. (1992) had stated a 1.3 MMSE score increase in oxiracetam compared to 0.2 in placebo.

### 5.3 Vascular Cognitive Impairment

A stroke/cerebral infarction can also lead to vascular cognitive impairment (VCI). Depending on the site of the lesion, multiple consequences can result, such as mood disturbances, memory problems/dementia, and cognitive decline. A prospective study including 80 participants was conducted on patients admitted to Dongguan City People's Hospital for post‐AIS cognitive impairment (PAISCI). Participants were assigned to either a study group or a control group. Following oxiracetam and butylphthalide treatment, MMSE scores increased significantly from 15.82 before treatment to 24.66, whereas in the control group, a minimal increase was observed from 15.73 (before treatment) to 18.63 (after treatment). A decline in TNF‐α (38.53 vs. 9.63), CRP (28.73 vs. 11.74), and IL‐6 (10.71 vs. 4.51) levels (pre‐treatment vs. post‐treatment) was also observed in the treatment group. These findings are important because TNF‐α and IL‐6 exacerbate VCI. Furthermore, high levels of IL‐6 also indicate how severe VCI is, by signifying low gray matter mass in the hippocampus. In conclusion, oxiracetam combined with butylphthalide demonstrated good efficacy in patients with post‐stroke VCI (Dong et al. 2023). VCI is also the second leading cause of dementia. Following the positive results of oxiracetam in previous human studies in patients with dementia, it can offer hope in patients with VCI‐induced dementia (Rozzini et al. 1992; Villardita et al. 1992).

### 5.4 Dementia Syndromes

Dementia is characterized by decreased cognitive ability severe enough to affect daily life tasks. Neurodegenerative dementias include Alzheimer's Disease (AD) and Lewy body dementia (Gale et al. 2018). AD is associated with severe cognitive decline due to the accumulation of amyloid‐β plaques extracellularly and tangles of tau protein intracellularly. A study found that oxiracetam inhibits activation of microglia induced by Aβ, reducing the release of pro‐inflammatory cytokines and nitric oxide. Oxiracetam also produces a protective effect on the hippocampus from indirect toxicity by microglial cells (Zhang et al. 2020).

A clinical trial by Rozzini et al. in participants with progressive dementia employed either 1600 mg of oxiracetam per day or a placebo in 96 patients. Outcomes were measured at 2 months, 6 months, and 12 months, and included reaction times (RTs) and several neurological tests. The study concluded that marked improvement in both these parameters was seen following oxiracetam therapy, while the placebo group had worsening symptoms and increased impairment of cognition (Rozzini et al. 1992).

Vascular dementia (VaD) is the second leading cause of dementia after AD in the elderly. In this, the impairment of blood flow leads to cognitive and behavioral decline. Neuronal lesions and neurodegeneration contribute to memory defects (Tarawneh and Holtzman 2012; Khan et al. 2016). As of yet, no drug has been approved for the treatment of VaD. In rats with bilateral common carotid artery occlusion (BCCAO), the imbalance between BCL‐2 (anti‐apoptotic) and BAX (pro‐apoptotic) contributes to the apoptosis of hippocampal neurons in VaD. As previously mentioned, oxiracetam can diffuse through the BBB and concentrate in the hippocampus. This imbalanced ratio of BCL‐2 to BAX was quickly reestablished after oxiracetam therapy in a clinical trial on VaD rats. Oxiracetam has also been shown to enhance AKT/mTOR signaling to exert protection against autophagy and apoptosis, proving that the drug could potentially enhance cognition and reduce neuronal damage (Xu et al. 2019).

## 6 Where Does Oxiracetam Stand Among Cognitive Enhancers?

The treatment spectrum for cognitive dysfunction is vast, starting from acetylcholinesterase (AChE) inhibitors such as donepezil, galantamine, and rivastigmine, used mainly in AD and also approved for vascular dementia. Unlike these agents that only inhibit AChE, oxiracetam also inhibits inflammatory cytokines, nitric oxide species, and the toxicity caused indirectly by the Aβ cascade, all of which are significant contributors to the disease process (Zhang et al. 2020; McCollum and Karlawish 2020). It also influences glutamate signaling in addition to the cholinergic pathway, offering balance to multifactorial neurotransmitter dysfunction (Yao et al. 2016). However, AChE inhibitors benefit from multiple larger‐scale Phase III trials (Mangalagiu et al. 2026), while oxiracetam trials until now have been limited to a smaller scale and population. Memantine is another drug employed in cognitive impairment, particularly moderate to severe AD and vascular dementia. It antagonizes the NMDA receptor, upregulating it and inhibiting excess glutamate (McCollum and Karlawish 2020). Since oxiracetam combines the properties of both AChE inhibitors (cholinergic) and memantine (glutamenergic), it could potentially be explored as an alternative therapy to memantine and donepezil combination therapy, minimizing their separate toxicities, medicinal expenses, dose frequency, and complications, such as GI disturbance, bradycardia, leg pain, and unusual dreams for AChE antagonists, and dizziness for memantine (McCollum and Karlawish 2020). Comparatively, oxiracetam has mild adverse effects (AEs) described in the following section.

In cognitive impairment due to TBI, two drugs are most commonly used, Amantadine and Methylphenidate, both of which have shown increased arousal, faster recovery, better attention span, and response rates (Barman et al. 2016). Originally introduced as an anti‐viral agent, amantadine has now been proven as a dopaminergic agent. However, most large‐scale trials on amantadine are heterogeneous in planning, participants, and results (Barra et al. 2022). Oxiracetam, by contrast, targets synaptic plasticity and glutamatergic modulation (McCollum and Karlawish 2020). While this mechanism aligns with the pathophysiology of diffuse axonal injury and synaptic disruption, direct head‐to‐head comparisons are lacking, and evidence supporting functional outcome improvement remains less robust than for amantadine in moderate‐to‐severe TBI.

Recently, disease‐modifying agents have also been used, especially in AD. Aducanumab, lecanemab, and donanemab are antibodies directed against Aβ bodies that prolong the cognitive decline in the initial phases of AD (Mangalagiu et al. 2026). Oxiracetam has similar anti‐Aβ properties (Zhang et al. 2020), along with the added effect of anti‐inflammatory and antioxidant actions, without the intense adverse effects of antibodies such as hypersensitivity (anaphylaxis or urticaria), cytopenias, hepatotoxicity, teratogenesis, cardiotoxicity, pulmonary effects, and skin reactions (Baldo 2022).

Compared to other racetams, including Piracetam, oxiracetam demonstrates greater potency in preclinical models and more consistent pharmacokinetic properties. However, the racetam class as a whole has historically suffered from heterogeneous clinical trial design and limited regulatory endorsement outside certain regions. Consequently, oxiracetam's perceived efficacy is often influenced by broader skepticism toward the class rather than drug‐specific data alone (Mangalagiu et al. 2026).

In VCI, PSCI, and VaD, no single pharmacologic agent has emerged as a definitive standard‐of‐care for cognitive restoration. In this therapeutic gap, oxiracetam could theoretically serve as an adjunctive neurorestorative agent. Nevertheless, recent large randomized trials have not conclusively demonstrated superiority over placebo, tempering enthusiasm for widespread adoption.

## 7 Safety and Tolerability

Adverse effects for L‐oxiracetam (4 g/day) were reported in 65.96% of patients. Specific treatment‐related side effects occurred in 9.36% of patients. The most common related effects included abnormal lab exams (6.81%), mental illness (0.85%), and heart disease (0.85%). On the other hand, the adverse effects of oxiracetam (6 g/day) were reported in 66.53% of patients. Notably, this group had a significantly higher rate of treatment‐related AEs (17.37%) compared to L‐oxiracetam (*p* = 0.049). They mostly included gastrointestinal diseases (2.54%) and skin/subcutaneous tissue diseases (2.12%), and anxiety (Liu et al. 2025; Zhang et al. 2024).

(S)‐oxiracetam has good tolerance and safety at all doses. No mortality, serious adverse events (SAE), dose‐dependent AE, or AE severe enough to cause discontinuation were reported. Most frequently reported AEs were mild and included an increase in the activated partial thromboplastin time (APTT) and triglyceride levels in (S)‐oxiracetam and elevated creatine kinase and ST‐segment abnormalities in oxiracetam. Mild accumulations of (S)‐oxiracetam were observed a week after administration. Trough concentrations remained constant from day 5 to day 7, indicating a steady state had been reached (Zhang et al. 2024).

Safety of oxiracetam even at 1600 mg/day was also confirmed up to 1 year of continuous treatment, even after follow‐up, with no serious side effects during the 1‐year period, and no reports of withdrawal effects were mentioned (Villardita et al. 1992). Gastrointestinal and systemic tolerability of oxiracetam was also categorized as “very good” when administered for 90 consecutive days (Falsaperla et al. 1990). In another study, oxiracetam was reported as having a favorable safety profile, described as mild and well‐tolerated (Li et al. 2023). Pulse rate and blood pressure remained unchanged, and no other clinically important changes were observed with oxiracetam in the blood (Saletu et al. 1985).

Oxiracetam is generally well tolerated, with side effects typically mild. Common adverse events include headaches, insomnia, gastrointestinal (GI) discomfort, restlessness, and nervousness.

## 8 Limitations and Future Directions

Earlier clinical investigations involving oxiracetam are mostly limited in sample size and underpowered. This reduces their ability to conclude clinically meaningful impact (Villardita et al. 1987). These early trials usually enrolled a small number of participants, and the outcomes were quite heterogeneous, making conclusions weak. Recently, trials with modest participant numbers are being conducted; a Korean multicenter trial enrolled 500 patients with PSCI, while the Phase III LOCATE trial included 590 patients with TBI. These studies collectively highlight the need for adequately powered trials to make the determination of efficacy and outcomes more reliable (Liu et al. 2025; Lim et al. 2026).

Beyond statistical power, the evidence base remains constrained by methodological limitations. Clustering by geography is also a major limitation in oxiracetam trials; 94.2% of participants in the LOCATE (Liu et al. 2025) trial were Han Chinese, while participants in Lim JS's study were exclusively from hospitals in South Korea. This regional concentration limits generalizability to Western and other non‐Asian populations. Moreover, the heterogeneity in cognitive outcome measures: LOTCA in the LOCATE trial, versus MMSE and CDR‐SB scores in the Korean study (Liu et al. 2025; Lim et al. 2026). This limits direct study comparisons, pooled analysis, and accurate estimation of treatment across a diverse patient population (Ndong 2026).

A further important limitation is the absence of biomarker‐driven stratification or mechanistic outcome assessment. As of yet, no clinical trial has utilized fluid or imaging biomarkers to guide or monitor the response to therapy. Future studies can employ objective indicators like serum neurofilament light chain that may aid in the identification of biologically responsive subgroups and neuroaxonal recovery measurement (Khalil et al. 2024). Aside from these trial limitations, one of the main challenges oxiracetam faces is that much of the evidence is derived from animal and cellular models that do not depict human networks and biology sufficiently. Historically speaking, multiple neuroprotective strategies showing strong preclinical efficacy have been unsuccessful in translation due to interspecies differences in neuronal and inflammatory signaling, injury diversity, and brain network organization (O'Collins et al. 2006). As a consequence, whether oxiracetam modulates synaptic plasticity, excitotoxicity, and inflammation in humans remains uncertain.

These limitations define key areas for researchers to consider for clinical trials in the future. Firstly, multicenter international randomized trials with a good sample size are important to synchronize outcomes and establish global generalizability. Follow‐up periods should be increased to evaluate long‐term cognitive advantages and safety outcomes. Secondly, targeted diagnostic measures that combine fluid biomarkers and neuroimaging can be helpful in identifying a specific group of patients who are most likely to benefit from oxiracetam (Khalil et al. 2024). Thirdly, the stereospecific advantages demonstrated by s‐oxiracetam represent enantiomer‐specific clinical research as the next step. Lastly, pairing up oxiracetam with other cognitive enhancers in combination therapies may produce increased functional recovery (Youn et al. 2023). Collectively, these strategies may help bridge the existing translational gap and support evidence‐based, personalized application of oxiracetam in cognitive disorders.

## 9 Conclusion

While oxiracetam shows impressive mechanisms, such as stimulating cholinergic fibers in the cerebral cortex and hippocampus, and improving synaptic plasticity, the currently available clinical trial results have been inconsistent so far. Oxiracetam is a generally safe and well‐tolerated drug, but currently lacks the definitive regulatory backing, such as from the FDA, and also lacks high‐quality confirmatory data that established drugs, such as acetylcholinesterase inhibitors, have. Based on our review, the results are heterogeneous due to the mixed trial results. However, its high safety profile makes it a plausible adjunctive drug, particularly where standard treatments fall short. More clinical trials need to be conducted to determine the clinical efficacy of oxiracetam, demonstrate the long‐term durability of cognitive benefits, and assess the drug's safety profile over very long follow‐up periods. Trials are also needed to determine whether the cognitive benefits observed in East Asian cohorts are generalizable to Western and other diverse, non‐Asian clinical populations.

## Author Contributions

**Hafsa Ali**: conceptualization; project administration; writing – original draft; writing – review and editing. **Syeda Maliha**: writing – original draft. **Sundas Ishtiaq**: writing – original draft. **Omer Al‐Karawi**: writing – original draft. **Mohammed Hammad Jaber Amin**: writing – review and editing.

## Funding

The authors have nothing to report.

## Conflicts of Interest

The authors declare no conflicts of interest.

## Ethics Statement

Ethical approval was not required for this study.

## Data Availability

Data sharing does not apply to this article as no new data were created or analyzed in this study.
